# Highly homologous proteins exert opposite biological activities by using different interaction interfaces

**DOI:** 10.1038/srep11629

**Published:** 2015-07-01

**Authors:** Anat Iosub Amir, Martijn van Rosmalen, Guy Mayer, Mario Lebendiker, Tsafi Danieli, Assaf Friedler

**Affiliations:** 1 Institute of Chemistry, The Hebrew University of Jerusalem, Safra Campus Givat Ram, Jerusalem 91904, Israel; 2 Wolfson Centre for Applied Structural Biology, The Hebrew University of Jerusalem, Safra Campus Givat Ram, Jerusalem 91904, Israel

## Abstract

We present a possible molecular basis for the opposite activity of two homologues proteins that bind similar ligands and show that this is achieved by fine-tuning of the interaction interface. The highly homologous ASPP proteins have opposite roles in regulating apoptosis: ASPP2 induces apoptosis while iASPP inhibits it. The ASPP proteins are regulated by an autoinhibitory interaction between their Ank-SH3 and Pro domains. We performed a detailed biophysical and molecular study of the Pro – Ank-SH3 interaction in iASPP and compared it to the interaction in ASPP2. We found that iASPP Pro is disordered and that the interaction sites are entirely different: iASPP Ank-SH3 binds iASPP Pro via its fourth Ank repeat and RT loop while ASPP2 Ank-SH3 binds ASPP2 Pro via its first Ank repeat and the n-src loop. It is possible that by using different moieties in the same interface, the proteins can have distinct and specific interactions resulting in differential regulation and ultimately different biological activities.

Protein–protein interactions are responsible for many cellular processes and many diseases are the result of impaired protein-protein interactions. In many cases, proteins use homologous domains to mediate their interactions with their partners. There are many cases where despite the high homology in the structure, the biological activity of such domains is different and even opposite. Here we show that fine-tuning of the binding interface underlies the molecular mechanism of this differential activity. Our model system is the ASPP (apoptosis stimulating proteins of p53) protein family, which plays a key role in regulating apoptosis. ASPP2 and ASPP1 activate apoptosis, while iASPP inhibits it^1^,^2^,^3^,^4^,^5^. ASPP2 is frequently downregulated in human cancers while iASPP is upregulated. Misregulation of the ASPP proteins is tightly associated with the malignancy of the tumor and with poor prognosis of the patients^1^,^6^,^7^,^8^,^9^,^10^,^11^,^12^.iASPP was identified as RelA-associated inhibitor (RAI), a 351 residues protein that binds and inhibits the transcriptional activity of the NFκB subunit p65^13^. Later it was discovered that RAI is the C-terminal part of an 828 residues protein termed iASPP (inhibitory member of the ASPP family)^14^ (Fig. 1a). iASPP has other important roles in cell regulation like inhibition of cell senescence and keratinocytes autophagy^15^,^16^.

The ASPP proteins share sequence and structural similarity at their C-termini, which contain four Ankyrin repeats and a Src Homology 3 (Ank-SH3) domains^7^,^17^ (Fig. 1a,b). The ASPP proteins also contain a proline rich (Pro) domain^2^,^14^. The 549 residues iASPP Pro is longer than the 226 residues ASPP2 Pro. We have previously shown that ASPP2 Pro is intrinsically disordered^18^. The iASPP N-terminal part of the Pro domain is responsible for its cytoplasmic localization^14^. ASPP2 contains an N-terminal domain with a ubiquitin like fold that iASPP lacks^2^,^19^.

The ASPP proteins interact with different apoptosis-related proteins such as the p53 protein family, Bcl2 and NFκB^2^,^7^,^13^,^20^,^21^,^22^,^23^,^24^. iASPP Pro interacts with iASPP Ank-SH3 in cells and phosphorylation of iASPP by B1/CDK1 on S84 and S113 inhibits this interaction^25^. Phosphorylation of iASPP results in its re-localization to the nucleus and inhibition of p53 activity. Inhibition of iASPP phosphorylation in melanoma cells restored p53 function and suppressed the melanoma growth^25^. ASPP2 is regulated by an interaction between its Ank-SH3 domains and Pro domain, which regulates the intermolecular interactions of ASPP2 with its different protein partners by an autoinhibitory mechanism^18^,^26^. The binding sites of p53, Bcl2 and NFκB to ASPP2 Ank-SH3 are different, while the binding sites of ASPP2 Pro to ASPP2 Ank-SH3 overlaps the binding sites to all three proteins^27^. Competition experiments showed that ASPP2 Pro competes with peptides from p53, Bcl2 and NFκB for binding ASPP2 Ank-SH3^18^,^26^. In *Helicobacter pylori* - infected cells, *H. pylori* protein CagA binds ASPP2, which results in ASPP2 binding to p53^28^,^29^. In these cells the interaction of ASPP2 with p53 is inhibited in the presence of ASPP2 726-782, which is derived from ASPP2 Pro, possibly because of this regulatory mechanism^30^.

Intrinsically disordered proteins (IDPs) or regions (IDRs) are highly flexible and lack a defined 3D structure^31^,^32^. IDPs and IDRs play crucial roles in many cellular processes such as transcriptional regulation, translation, recognition and signal transduction^31^,^32^. IDPs and IDRs can bind their partners with high specificity but low affinity^32^ and their binding to their partners is often regulated by post translational modifications such as acetylation, phosphorylation and methylation^31^. In many proteins that are regulated by auto-inhibition, the inhibitory region is highly disordered and has many phosphorylation sites^33^.

Despite the sequential and structural homology between their Ank-SH3 domains, the ASPP proteins have opposite activities in regulating apoptosis. The N-terminal domain that is unique to ASPP2 is not responsible for this difference^34^,^35^. To gain insight into the molecular mechanism behind this difference, we performed a detailed biophysical and molecular study of the Pro – Ank-SH3 interaction in iASPP and compared it to the interaction in ASPP2. We developed new protocols for expressing and purifying iASPP Pro. Using biophysical and computational methods we show that iASPP Pro is disordered, like ASPP2 Pro, and that the purified iASPP Pro and iASPP Ank-SH3 interact with each other *in vitro*. Peptide array screening revealed the exact binding sites between the iASPP domains. Our results show that the Pro-binding regions in iASPP Ank-SH3 are different than the Pro-binding regions in ASPP2 Ank-SH3^18^, revealing selectivity and specificity between the ASPP proteins. This sheds light on the molecular basis for the difference in activity between the ASPP proteins.

## Results

### Development of a new protocol protocol for the expression and purification of iASPP Pro

**Figure 2:**
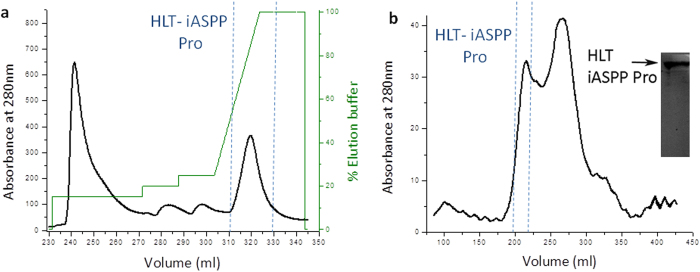
Purification of HLT-iASPP Pro. (**a**) Elution profile from the Nickel Sepharose column using imidazole gradient (green) as monitored by UV absorbance at 280 nm; (**b**) Elution profile from the size exclusion chromatography sephacryl S200 500 ml column as monitored by UV absorbance at 280 nm. Inset: Coomassie staining of the SDS-PAGE gel of the purified HLT-iASPP Pro collected from the size exclusion chromatography column, Mw = 75.9 kDa, marked with an arrow.

HLT-iASPP Pro was expressed in *E.coli* Rosetta2 (Novagen) as described in materials and methods. HLT-iASPP Pro initially showed a high tendency to aggregate. Following the screening of conditions as described in materials and methods^36^, aggregation was minimized in 50 mM phosphate buffer pH = 7, 300 mM NaCl, 10% glycerol and 0.001% Tween 20. HLT-iASPP Pro was purified in two steps, including affinity chromatography using a Nickel Sepharose column (Fig. 2a) followed by size exclusion chromatography (Fig. 2b). An imidazole gradient was used for eluting HLT-iASPP Pro from the Nickel column. HLT-iASPP Pro eluted in 100% elution buffer containing 300 mM Imidazole. Unspecific bound contaminations eluted in 10%–20% elution buffer. The 602 residues iASPP-Pro was expressed with it truncated forms. These impurities, which eluted with the full protein from the Nickel column, were separated from the full length protein by using size exclusion chromatography. The full protein eluted first from the size exclusion column and was successfully separated from its truncated forms. We did not cleave the HLT tag to avoid the aggregation of the protein. The final concentration of HLT-iASPP Pro was 15 μM.

### iASPP Pro is intrinsically disordered

**Figure 3:**
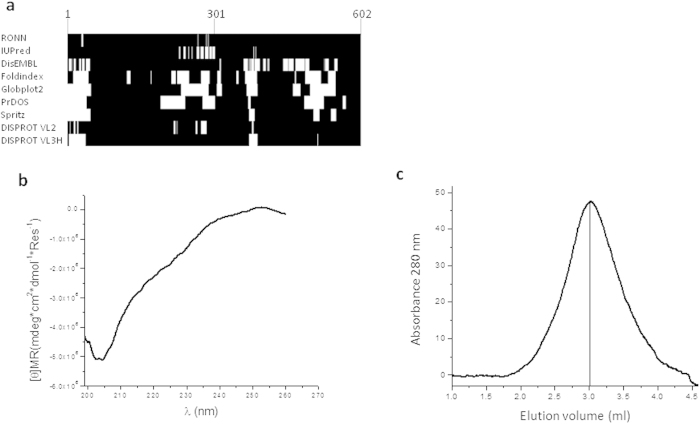
iASPP Pro is intrinsically disordered. (**a**) Disorder prediction of iASPP Pro (1-602). Black- residues predicted to be disordered. Each row represents a different server; (**b**) CD spectrum of HLT-iASPP Pro. The spectrum did not show any characteristic secondary structure; (**c**) Analytical size exclusion chromatography results for purified HLT-iASPP Pro. The calculated Mw is 75.9 kDa but the protein eluted at a volume corresponding to Mw of ~250 kDa, supporting that it is disordered.

To characterize the structural properties of iASPP Pro, we used a combination of computational and experimental tools. Several disorder prediction servers predicted iASPP Pro (iASPP 1-602) to be mostly disordered (Fig. 3a). The CD spectra of purified HLT-iASPP Pro (Fig. 3b) did not show any characteristic secondary structure, indicating intrinsic disorder. HLT-iASPP Pro (MW = 75.9 kDa) eluted in size exclusion chromatography experiments as a wide peak with an elution volume corresponding to a 250 kDa globular protein (Fig. 3c). This further indicates that iASPP Pro is disordered since disordered proteins elute earlier than globular proteins of the same MW, due to their extended unfolded nature. Our results imply that iASPP Pro is intrinsically disordered, like ASPP2 Pro.

### iASPP Ank-SH3 binds iASPP Pro *in vitro*

**Figure 4:**
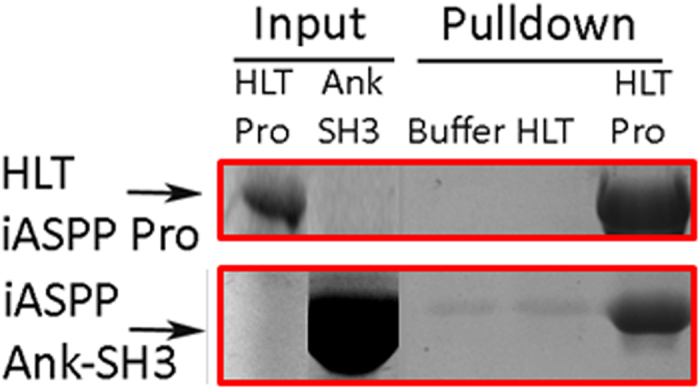
HLT-iASPP Pro interacts with iASPP Ank-SH3 *in vitro*. Nickel affinity pulldown of iASPP Ank-SH3 by HLT-iASPP Pro, SDS PAGE gel coomassie staining results. Nickel-NTA beads were incubated with HLT-iASPP Pro or with buffer or HLT fusion domain alone, for 1 h at 4 °C. The samples were centrifuged and then incubated with iASPP Ank-SH3 for 2 h at 4 °C. The samples were centrifuged again and the beads were washed 3 times. The beads were eluted by boiling them in SDS solution. Ank-SH3 eluted from the beads only in the presence of HLT-iASPP Pro.

After purifying the recombinant HLT-iASPP Pro and iASPP Ank-SH3 we tested whether the recombinant proteins interact using Nickel affinity pulldown assay. Nickel-NTA beads were incubated for one hour with HLT-iASPP Pro or with buffer or HLT alone. Then the beads were incubated with iASPP Ank-SH3 for two hours. After three washes the proteins were eluted from the Nickel-NTA beads. iASPP Ank-SH3 was retrieved by Nickel-NTA beads that were incubated with HLT-iASPP Pro but not by Nickel-NTA beads that were incubated with buffer or HLT (Fig. 4) indicating that both iASPP domains interact with each other.

### Mapping the binding sites between the iASPP domains

**Figure 5:**
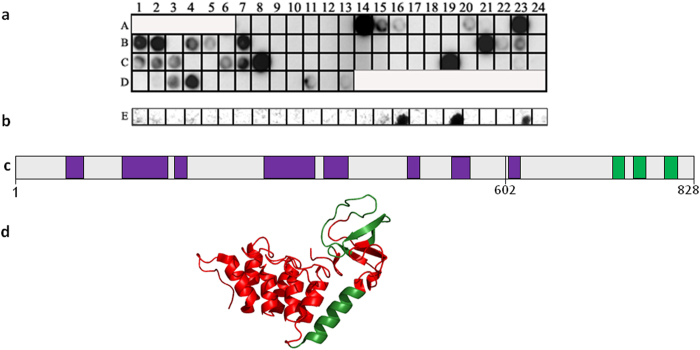
The binding sites between the iASPP domains: peptide array screening. (**a**) Binding of HLT-iASPP Ank-SH3 to an array of peptides derived from iASPP Pro at IS = 150 mM. Dark spots indicate binding peptides. For peptide sequences see Table 1; (**b**) Binding of HLT-iASPP Pro to an array of peptides derived from iASPP Ank-SH3. Dark spots indicate binding peptides. For peptide sequences see Table 2; (**c**) The binding interface between the iASPP domains: The HLT-iASPP Ank-SH3 binding regions in iASPP Pro are colored purple. The HLT-iASPP Pro binding regions in iASPP Ank-SH3 are colored green; (**d**) The iASPP Pro binding regions in iASPP Ank-SH3, labeled in green on the crystal structure of iASPP Ank-SH3. PDB: 2VGE. The figure was generated using PyMOL (The PyMOL Molecular Graphics System, Version 1.3. Schrödinger, LLC).

To map the sites in the iASPP domains that mediate the interactions, HLT-iASPP Ank-SH3 was screened for binding an array comprising 79 partly overlapping 15-residue peptides derived from iASPP Pro^37^ (Table S1). HLT-iASPP Ank-SH3 bound 19 peptides with a strong signal (Table 1, Fig. 5a,c) at an ionic strength (IS) of 150 mM. The binding peptides were derived from iASPP Pro regions spanning residues 60-82, 132-186, 196-210, 308-370, 380-410, 484-498, 540-562, and 604-618. HLT-iASPP Pro was screened for binding an array comprising 24 partly overlapping 15-residue peptides derived from iASPP Ank-SH3 (Table S1). HLT-iASPP Pro bound three peptides derived from iASPP Ank-SH3 residues 739-753, 764-778, and 800-814 (Figs 5b–d, Table 2).

**Figure 6:**
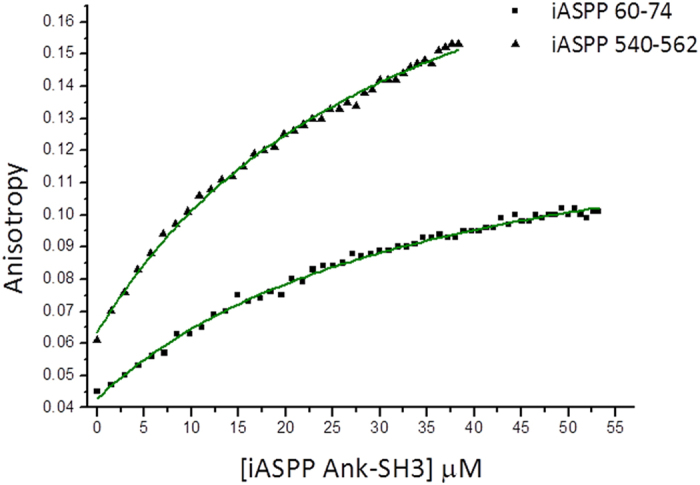
Binding of iASPP Ank-SH3 to the FL-iASPP derived peptides. Fluorescence anisotropy results: 250 μM iASPP Ank-SH3 were titrated into fluorescein labeled iASPP derived peptides. *K*_*d*_ for iASPP 60-74 was found to be 35 ± 2 μM (square) while *K*_*d*_ for iASPP 540-562 was found to be 34 ± 2 μM (triangle).

**Figure 7:**
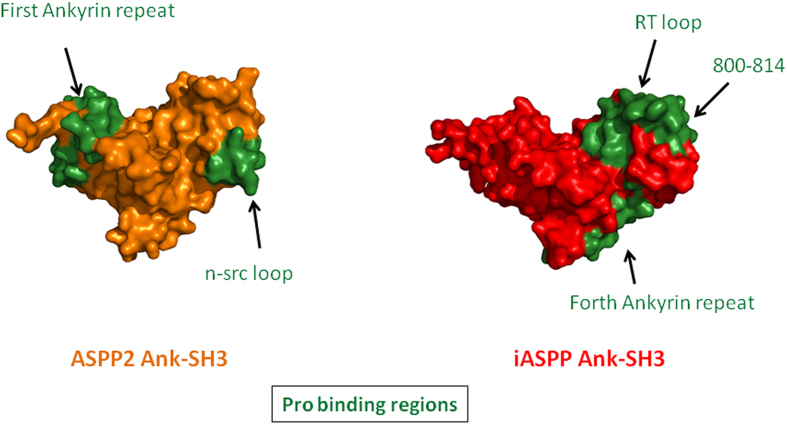
The Ank-SH3 domains of iASPP and ASPP2 bind their Pro domains through distinct interfaces. iASPP Ank-SH3 (red) binds iASPP Pro using its fourth ankyrin repeat, RT loop and residues 800-814 while ASPP2 Ank-SH3 (orange) binds ASPP2 Pro via its first ankyrin repeat and n-src loop. Green-Pro binding regions. iASPP PDB: 2VGE, ASPP2 PDB: 4A63. All Figures were generated using PyMOL (The PyMOL Molecular Graphics System, Version 1.3. Schrödinger, LLC).

We quantified the interactions of the iASPP Pro derived peptides with iASPP Ank-SH3 using fluorescence anisotropy. The two tightest binding peptides were iASPP 60-74, which bound iASPP Ank-SH3 with *K*_*d*_ of 35 ± 2 μM, and iASPP 540-562, which bound iASPP Ank-SH3 with *K*_*d*_ of 34 ± 2 μM (Fig. 6). iASPP Ank-SH3 binding to iASPP 132-146, iASPP 172-186, iASPP 396-410 and iASPP 484-498 was observed but was too weak to quantify. iASPP 68-82, iASPP 156-170, iASPP 196-210, iASPP 308-322 and iASPP 324-338 showed no binding to iASPP Ank-SH3. iASPP 83-90, which includes the iASPP phosphorylation site S84^25^, did not bind iASPP Ank-SH3 in the peptide array or in fluorescence anisotropy experiments. Altogether, our results indicate that different interfaces on the Ank-SH3 domains of the ASPP proteins mediate their interactions with their Pro domains (Fig. 7). Three main regions of iASPP Ank-SH3 interact with iASPP Pro. These regions include the fourth Ank repeat (iASPP 739-753), the RT loop (iASPP 764-778) and the C-terminal residues 800-814. On the other hand, we have previously shown that ASPP2 Ank-SH3 binds ASPP2 Pro through the first Ankyrin repeat (ASPP2 931-961) and the n-src loop (ASPP2 1083-1096)^18^.

## Discussion

Despite the sequential and structural homology between their Ank-SH3 and Pro domains, the ASPP proteins have opposite activities in regulating apoptosis. To gain insight into the molecular mechanism behind this difference, we performed a detailed biophysical and molecular study of the Pro – Ank-SH3 interaction in iASPP and compared it to the interaction in ASPP2. In order to enable these quantitative biophysical studies, we developed for the first time a protocol for expressing and purifying a stable recombinant full-length iASPP Pro at concentrations sufficient for biophysical studies. We also made some modifications to the known protocol for producing recombinant iASPP Ank-SH3, mainly using the HLT tag^7^. Having the two recombinant proteins in hand at high purity and concentrations enabled us to show that iASPP Pro is intrinsically disordered like ASPP2 Pro^18^. Unlike other IDPs, iASPP Pro does not mediate interactions with other partner proteins^32^.

### The binding interface between the iASPP domains

Our results show that iASPP Ank-SH3 and the full iASPP Pro 1-602 interact *in vitro*. It was shown before that the interaction of iASPP Pro with iASPP Ank-SH3 in cells is inhibited by phosphorylation of iASPP Pro on S84 and S113 by B1/CDK1^25^. However it is not clear from the cellular studies or from our *in vitro* studies if the interaction between the iASPP domains is intramolecular or intermolecular^18^,^25^. The fact that iASPP Pro is intrinsically disordered might support the intramolecular possibility because many proteins that are regulated by autoinhibitory mechanism have intrinsically disordered regulatory domains. Many of these regulatory domains undergo alternative splicing, such as in the ASPP proteins, and have many phosphorylation sites^33^,^38^. In any case, the final regulatory outcome is the same regardless of whether the domain-domain interaction is intramolecular or involves dimerization.

iASPP 1-478 and specifically iASPP 1-240 but not iASPP 249-482 were previously shown to bind iASPP Ank-SH3 in a pull down assay^25^. We further mapped this interaction using peptide arrays and identified the exact regions in iASPP Pro that bind iASPP Ank-SH3. The two tightest binding peptides were iASPP 60-74, which is derived from the binding region iASPP 1-240, with *K*_*d*_ of 35 ± 2 μM, and iASPP 540-562, which represents a previously unknown binding site for iASPP Ank-SH3 in iASPP Pro, with *K*_*d*_ of 34 ± 2 μM. iASPP 60-74 is located close to the iASPP phosphorylation sites, indicating possible regulation by phosphorylation. Other iASPP 1-240 derived peptides that bound iASPP Ank-SH3 in the peptide array are not derived from iASPP phosphorylation sites or the sequence between these two sites (Table 1). Peptides derived from iASPP 249-482, and specifically iASPP 308-410, also bound iASPP Ank-SH3 in the peptide array experiment (Table 1), but showed no binding or the binding was too weak to quantify. iASPP 616-623 is also a part of the interaction interface: it bound iASPP Ank-SH3 with a *K*_*d*_ of 45 μM in a previous ITC experiment^7^ and its slightly overlapping peptide iASPP 604-618 bound iASPP Ank-SH3 in our peptide array experiments. Altogether, the Ank-SH3 binding regions in iASPP Pro are spread along the full length disordered Pro domain. Two of the tightest binding peptides, iASPP 60-74 and iASPP 616-623, are located at the two termini of the Pro domain. The affinities of the peptides derived from the Pro domain to the Ank-SH3 domain are low, as is expected for disordered domains^32^,^38^. The affinity of SH3 domains to their proline rich ligands is also known to be weak^39^,^40^. Mutations in iASPP Ank-SH3 showed that iASPP Ank-SH3 N813 and Y814 and to a lesser extent T722 and L724 are important for binding iASPP 1-240^25^. Here we found that the peptide iASPP 800-814 that includes two of these residues bound iASPP Pro in the peptide array. iASPP 739-753, which is derived from the fourth ankyrin repeat, and iASPP 764-778, which is derived from the RT loop in the SH3 domain, also bound iASPP Pro.

### The binding interfaces in iASPP vs. ASPP2

Comparing the Pro - Ank-SH3 interaction interfaces in iASPP and ASPP2^18^ revealed distinct and specific binding sites, which are totally different from each other. iASPP Pro binds the fourth Ank repeat (iASPP 739-753), RT loop (iASPP 764-778) and C-terminal residues (iASPP 800-814) of iASPP Ank-SH3. ASPP2 Pro binds the first Ank-repeat (ASPP2 931-961) and n-src loop (ASPP2 1083-1096) of ASPP2 Ank-SH3^18^ (Fig. 7). The interface in iASPP Pro that mediates the interaction with its Ank-SH3 domain is much larger than the corresponding interface in ASPP2 Pro, probably because iASPP Pro is more than twice longer than ASPP2 Pro.

Our results can explain previous data reported in the literature regarding the interactions of ASPP2 and iASPP with p53. Energy assessment for p53 Core domain (p53CD) complexes with the Ank-SH3 domains of ASPP2 and iASPP^27^ showed the complex of p53CD with iASPP Ank-SH3 had a higher interaction energy than the complex of p53CD with ASPP2 Ank-SH3^17^. This indicates that the binding mode is indeed different. Previous NMR experiments showed that the SH3 domains of iASPP and ASPP2 have a very similar binding interface with the Proline rich and core domains of p53 (p53 Pro + CD), which includes both the RT and n-src loops, while ASPP2 interacts with p53 Pro + CD also via the loops between Ank repeats 2-3 and 3-4^41^. The crystal structure of ASPP2 Ank-SH3 complex with p53CD shows that the fourth Ank repeat and the RT and n-src loops of ASPP2 mediate this interaction^17^. However, the ASPP2 derived n-src loop peptide bound p53CD much tighter than peptides derived from the fourth Ank repeat and the RT loop of ASPP2^42^. Docking studies performed for the iASPP Ank-SH3 - p53CD complex showed that the interaction between them is mediated mostly by the RT loop of iASPP. Mutational studies based on these results showed that only mutations in the iASPP RT loop, but not mutations in the n-Src loop, abolished the inhibitory effect of iASPP Ank-SH3 on p53-mediated expression of apoptosis-related genes^43^. These results are in line with our previous results, showing that the n-Src loop but not the RT loop is the binding site of ASPP2 Ank-SH3 to its Pro domain, while it is the opposite for the iASPP Ank-SH3- Pro interaction. This may indicate that the n-Src loop is a general binding interface of ASPP2, while the RT loop is a general binding interface of iASPP.

### The possible molecular basis for the opposite activity of iASPP and ASPP2

Previous studies performed in our lab suggested that an important parameter in the molecular basis for the different activity of the ASPP proteins is the different electrostatic potential of the surfaces of their Ank-SH3 domains. The binding interface of ASPP2 has a higher negative charge compared to its homologous surface in iASPP^27^. Examining the sequences of the ASPP proteins revealed that indeed the first Ank repeat and n-src loop of ASPP2 are more negatively charged than these areas in iASPP, while the fourth Ank repeat of iASPP is more negatively charged than this area in ASPP2. The RT loops of both ASPP proteins have the same charge, but there is a difference in the location of their negatively charged residues.

Other examples of highly homologous proteins that bind differentially to their ligands were reported. Sequence variability in the RT and n-src loops in SH3 domains can lead to binding specificity, for example the HIV-1 Nef protein interacts only with the SH3 domain of the Hck protein but not with similar src family members due to one different residue in its RT loop^44^. There are other examples of structurally homologous proteins that bind similar ligands in different ways, but the activity of the proteins is not opposite. For example the retinoid transport proteins bind and transport retinoids in different sub-cellular areas and tissues. Although they have very similar architectures they bind the retinoids in different ways^45^.

Our results suggest that fine-tuning of the interaction interface can lead to different and even opposite activities of highly homologous proteins. By using different moieties in the same interface, the proteins can have distinct and specific interactions resulting in differential regulation and ultimately different biological activities. Our results also shed light on the possible molecular basis for the difference in activity between the ASPP proteins, where iASPP is anti-apoptotic while ASPP2 is pro-apoptotic. The different binding interfaces to the regulatory Pro domains in these highly similar structures may be part of the reasons for the different biological activities. As misregulation of the ASPP proteins is responsible for many cancerous transformations, understanding the molecular basis for the opposite activity of the ASPP proteins will provide the basis for developing future anti-cancer lead compounds that inhibit iASPP or mimic the apoptotic activity of ASPP2.

## Methods

### Plasmids preparation

The plasmid containing iASPP 1-828 was a kind gift of Prof. Xin Lu, Ludwig Institute for Cancer Research, Oxford, UK. DNAs encoding fragments of iASPP were amplified by PCR using primers introducing an upstream EcoRI restriction site and a downstream NotIrestriction site: (1) iASPP Pro 1-602, 5´-GTGTACCGGAATTCAAGACAGCGAGGCATTCCAGAGC-3´, 5´-GTAAGAATGCGGCCGCCTACGGCTGCTCTGTGGGC-3´; (2) iASPP Ank-SH3 603-828, 5´-GTG TAC CGG AAT TCA ACA GAG CAT GGA GAT GCG CTC TG-3´, 5´-GTA AGA ATG CGG CCG CCT AGA CTT TAC TCC TTT GAG GCT TCA CCC TG-3´. PCR products were separated on 1% agarose gel and purified using GFXTm PCR DNA and gel band purification kit (Amersham). The PCR products were heated to 97 °C, cooled down to 37 °C and then cleaved by EcoRI and NotI (New England Biolabs) and purified again. The insert fragments were ligated (ligation master mix TAKARA) into the ampicillin resistant pET-Based vector pHis parallel 2HLT (HLT tag- The lipoyl domain fusion tag containing the N-terminus His tag and an optimized Tobacco Etch Virus (TEV) protease cleavage site^36^). The ligation reaction product was transformed into competent *Escherichia coli* Stable3 bacteria (Invitrogen) and bacterial colonies were screened for the presence of the gene using PCR. Positive colonies were further verified by sequencing. The analyzed sequence of the vector containing domains of the iASPP gene was compared and found identical to the iASPP sequence from Swiss-Prot entry Q8WUF5 (IASPP_HUMAN).

### Protein expression and purification

#### HLT-iASPP Pro

was expressed in *Escherichia coli* Rosetta2 (Novagen). Bacteria were grown in 2xYT medium containing 1% glucose at 37 °C. Induction was performed at A_600 nm_ = 0.6 with 0.4 mM Isopropyl β-D-1-thiogalactopyranoside (IPTG). Cells were harvested after 16 h of incubation at 17 °C. Bacterial cells expressing HLT-iASPP Pro were lysed using a Microfluidizer (Microfluidics). HLT-iASPP Pro showed a high tendency to aggregate. To overcome this, we screened different expression conditions, which included different buffers, NaCl concentrations and additives that included Arginine, Glutamate, Tween 20, Tween 80, zwitergent, trehalose and Trimethylamine N-oxide. Aggregation of HLT-iASPP Pro was minimized in phosphate buffer pH = 7, 300 mM NaCl, 10% glycerol and in the presence of 0.001% Tween 20. HLT-iASPP Pro was lysed in buffer A (50 mM Phosphate pH = 7.0, 0.3 M NaCl, 10% glycerol) containing 1% Tween20, lysozyme, DNase, 0.2 mM MgSO_4_, and 1:200 protease inhibitor P-8849 (Sigma) and purified using an Äkta Explorer FPLC system (GE Healthcare). The soluble fraction was purified using a Nickel Sepharose FF 4 ml column. Elution was performed using an imidzole gradient (Buffer A + 0.001% Tween 20 and 300 mM imidazole). HLT-iASPP Pro was further purified using size exclusion chromatography on a Sephacryl S200, 500 ml column. Elution was performed with buffer A + 0.001% Tween 20, which was also used as the storage buffer. The final concentration of HLT-iASPP Pro was 15 μM.

#### HLT-iASPP Ank-SH3

was expressed in *E.coli* HMS 174 (Novagen). Bacteria were grown in 2xYT medium containing 1% glucose at 37 °C. Induction was done at A_600 nm_ = 0.6 with 0.1 mM IPTG for HLT-iASPP Ank-SH3. Cells were harvested after 16 h of incubation at 23 °C. iASPP Ank-SH3 was lysed using a Microfluidizer (Microfluidics) in buffer B (20 mM Tris-HCl pH = 8.0, 0.5 M NaCl, 10% glycerol, 5 mM β-mercaptoethanol) containing lysozyme, DNase, 0.2 mM MgSO_4_, and 1:200 protease inhibitor P-8849 (Sigma). iASPP Ank-SH3 was purified using an Äkta Explorer FPLC system (GE Healthcare). The soluble fraction was purified using a Nickel Sepharose FF 8 ml column. Elution was performed using an imidzole gradient. HLT-iASPP Ank-SH3 eluted in 100% elution buffer (containing buffer B + 250 mM imidazole). HLT-iASPP Ank-SH3 was further purified using size exclusion chromatography on a Sephacryl S100 column. Elution was performed with 20 mM Phosphate pH = 7.0, 0.15 M NaCl, 5 mM βMe, 10% glycerol and 0.02% NaN_3_, which was also used as the storage buffer. For cleaving the HLT tag from HLT-iASPP Ank-SH3, buffer B + 35 mM imidazole was used. HLT-iASPP Ank-SH3 was incubated for 16 h at 4 °C with Hisx6-TEV protease at a 20:1 (HLT-iASPP Ank-SH3:Hisx6-TEV) ratio. The Hisx6-tagged TEV protease, HLT-tag, impurities and any residual uncleaved HLT-iASPP Ank-SH3 were removed by an additional Nickel column step using Sepharose FF 4 ml column. The cleaved iASPP Ank-SH3 did not bind to the column and was further purified using size exclusion chromatography on a Sephacryl S100 column as described for HLT-iASPP Ank-SH3.

### Disorder predictions

The following servers were used for disorder prediction of iASPP Pro: RONN^46^, IUPred^47^, DisEMBL^48^, Foldindex^49^, GlobPlot2^50^, PrDOS^51^, Spritz^52^ and DisPROT^53^. In all cases, iASPP Pro 1-602 (Swiss-Prot entry Q8WUF5) was subjected to disorder prediction using default server parameters.

### Circular Dichroism (CD)

CD spectra of HLT-iASPP Pro were recorded using a J-810 spectropolarimeter (Jasco) in a 0.1 cm quartz cuvette for far-UV CD spectroscopy, in a spectral range of 197 nm to 260 nm. 3 μM HLT- iASPP Pro was dissolved in 50 mM Phosphate buffer pH = 7.0, 300 mM NaCl, 10% glycerol, 0.02% NaN3 and 0.001% Tween 20.

### Nickel affinity Pulldown assay

30 μl Nickel-NTA beads (Qiagen) were incubated with: (1) 500 μl buffer C (50 mM Phosphate buffer pH = 7.0, 300 mM NaCl, 10% glycerol, 0.02% NaN_3_ and 0.001% Tween 20); (2) 250 μl 31 μM HLT; (3) 2 ml 1.7 μM HLT-iASPP Pro (in buffer C) for 1 hour with gentle mixing at 4 °C and in the presence of 40 mM imidazole. The samples were centrifuged (2 min 3500 RPM), and washed with buffer C (5 min, 4 °C, gentle mixing). 80 μl of 80 μM iASPP Ank-SH3 in 20 mM Phosphate buffer pH = 7.0, 150 mM NaCl, 5mM βMe, 10% glycerol, 0.02% NaN3, 15 mM imidazole were added to the three samples. For diluting iASPP Ank-SH3, each sample was added to 1 ml 25 mM phosphate buffer pH = 7.0, 10% glycerol, 25 mM Imidazole and 100 mM NaCl. The Nickel-NTA beads and iASPP Ank-SH3 were gently mixed for 2 hours at 4 °C. The samples were centrifuged and the supernatants were collected. The samples were washed three times with 1 ml buffer C and the third washes were kept. The beads were eluted by boiling them in SDS sample solution. Samples were analyzed on a 10% SDS-PAGE gel.

### Peptide array screening

The CelluSpots^TM^ peptide micro-arrays were synthesized by INTAVIS Bioanalytical Instruments AG, Köln, Germany. The 15 residue peptides were acetylated at their N-termini and attached to a cellulose membrane via their C termini through an amide bond. For screening the binding of the array to HLT-iASPP Ank-SH3 the array was first washed for 4 h at room temperature with 50 mM Tris-HCl pH = 8 at an ionic strength (IS) of 150 mM adjusted by NaCl, 0.05% Tween20 and 2.5% (W/V) skimmed milk (buffer D) for blocking unspecific binding. HLT-iASPP Ank-SH3 was dissolved in 20 mM Phosphate buffer pH = 7.0 IS = 150 mM, 5 mM βME, 10% glycerol and 0.02% NaN_3_ and 2.5% skimmed milk. 5 ml of 5 μM of the protein were incubated with the arrays at 4 °C with shaking overnight. After three washes with TBST, the array was incubated with anti His HRP conjugated antibody at room temperature for 1 hour. The antibody was dissolved in buffer D. Then the array was washed again three times with TBST. Immunodetection was performed using chemiluminiscence (ECL reagents). Peptide array screening for binding HLT-iASPP Pro was also preformed as above with change of the proteins buffer. HLT-iASPP Pro was dissolved in 50 mM Phosphate buffer pH = 7.0, 10% glycerol, 0.02% NaN3, 0.001% Tween 20, IS = 150 mM and 2.5% skimmed milk.

### Peptide synthesis, labeling and purification

Peptides were synthesized on a Liberty Microwave Assisted Peptide Synthesizer (CEM) using standard Fmoc chemistry and HOBT/HBTU as coupling reagents. Trp was added at the N-termini of the peptides, when required, for measuring the peptide concentration using UV spectroscopy. The peptides were labeled with 5(6)-carboxyfluorescein at their N termini as described^54^ and cleaved from the resin as described^55^. The peptides were purified on a MERCK-Hitachi HPLC using a reverse-phase C8 preparative column with a gradient of ACN/TDW. MALDI TOF mass spectrometry and analytical HPLC were used to verify the identity and purity of the peptides.

### Fluorescence anisotropy-binding studies

Binding of the iASPP Pro-derived peptides to iASPP Ank-SH3 was measured by fluorescence anisotropy using a PerkinElmer Life Sciences LS-50b spectrofluorimeter equipped with a Hamilton microlab M dispenser. Titration of iASPP Ank-SH3 into iASPP Pro derived fluorescein-labeled (FL) peptides was performed at 10 °C in 20 mM Hepes buffer, pH 7.3, 43 mM NaCl (total ionic strength = 50 mM), 10% glycerol and 5 mM β-mercaptoethanol. The excitation wavelength was set at 480 nm and the emission measured at 530 nm. The labeled peptide, dissolved in 1 ml buffer to a final concentration of 100 nM, was placed in the cuvette. 250 μM iASPP Ank-SH3 was placed in the dispenser and aliquots of 10–20 μl were added at 1.5 min intervals. The solution was then stirred for 30 sec, and the fluorescence and anisotropy were measured. Data were analyzed using the program Origin8 (OriginLab) and were fit to 1:1 binding model:


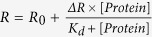
 (R- measured fluorescence anisotropy, R_0_- anisotropy value corresponding to the free peptide, ∆R- amplitude of the fluorescence anisotropy change, [Protein]- protein concentration, *K*_*d*_- dissociation constant).

## Additional Information

**How to cite this article**: Iosub Amir, A. *et al.* Highly homologous proteins exert opposite biological activities by using different interaction interfaces. *Sci. Rep.* **5**, 11629; doi: 10.1038/srep11629 (2015).

## Supplementary Material

Supplementary Information

## Figures and Tables

**Figure 1:**
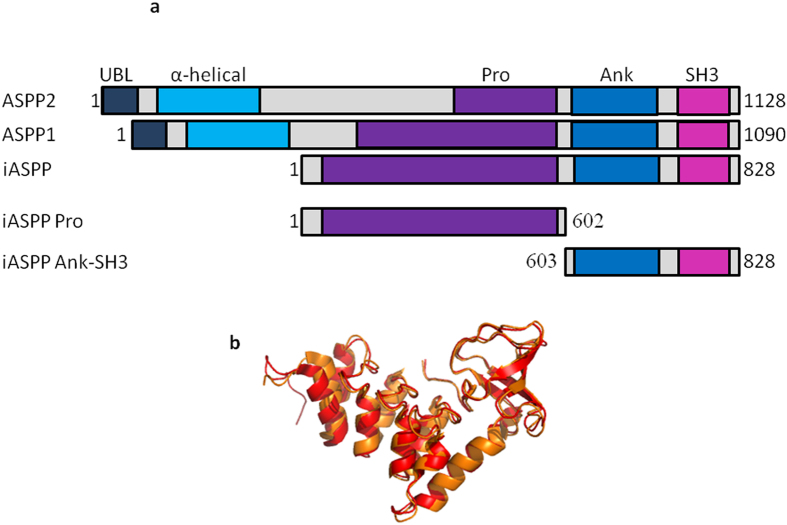
The ASPP protein family. (**a**) All the ASPP protein family members contain a Proline rich (Pro) domain, four ankyrin repeats (Ank) and an SH3 domain. ASPP2 and ASPP1 also contain a putative α-helical domain at their N- termini. The N-terminal part of ASPP2 has the structure of a β-Grasp ubiquitin-like fold (UBL)^19^. The iASPP fragments used in this study are iASPP Pro and iASPP Ank-SH3; (**b**) Backbone alignment of the crystal structures of ASPP2 Ank-SH3 (920-1121), PDB:4A63^56^ (orange) and iASPP Ank-SH3 (607-828), PDB: 2VGE^7^ (red). The alignment shows the structure similarity between ASPP2 Ank-SH3 and iASPP Ank-SH3. Figure was made using Pymol (The PyMOL Molecular Graphics System, Version 1.3. Schrödinger, LLC).

**Table 1:** The iASPP Ank-SH3 binding regions in iASPP Pro: peptide array results.

Peptide Spot	iASPP Pro residues	peptide sequence
A14	60–74	QAGPPSRPPRYSSSS
A15	68–82	PRYSSSSIPEPFGSR
A23	132–146	YGSLDRATSPRPRAF
B1	148–162	GAGSSLGRAPSPRPG
B2	156–170	APSPRPGPGPLRQQG
B4	172–186	PTPFDFLGRAGSPRG
B7	196–210	FFPERGPSPRPPATA
B21	308–322	TLPRNYKVSPLASDR
B23	324–338	SDAGSYRRSLGSAGP
C1	340-354	GTLPRSWQPVSRIPM
C2	348–362	PVSRIPMPPSSPQPR
C3	356–370	PSSPQPRGAPRQRPI
C6	380-394	NAFWEHGASRAMLPG
C7	388–402	SRAMLPGSPLFTRAP
C8	396–410	PLFTRAPPPKLQPQP
C19	484–498	VARPLSPTRLQPALP
D3	540–554	KKQYQQIISRLFHRH
D4	548–562	SRLFHRHGGPGPGGP
D11	604–618	SMEMRSVLRKAGSPR

**Table 2:** The iASPP Pro binding regions in iASPP Ank-SH3: peptide array results.

Peptide Spot	iASPP Ank-SH3 residues	peptide sequence
E16	739–753	EGYADCATYLADVEQ
E19	764–778	YALWDYSAEFGDELS
E23	800–814	WAALHGQEGYVPRNY
