# Supplementary material for: Highly homologous proteins exert opposite biological activities by using different interaction interfaces
Source: Sci Rep. 2015 Jul 1;5:11629. doi: 10.1038/srep11629 (PMC4486954; doi:10.1038/srep11629)
Supplement: Supplementary Information [file srep11629-s1.doc]

**Highly homologous proteins exert opposite biological activities by using different interaction interfaces**

Anat Iosub Amir^1^, Martijn van Rosmalen^1^, Guy Mayer^1^, Mario Lebendiker^2^, Tsafi Danieli^2^ and Assaf Friedler^*1^

^1^Institute of Chemistry and ^2^Wolfson Centre for Applied Structural Biology, The Hebrew University of Jerusalem, Safra Campus Givat Ram, Jerusalem 91904, Israel

*corresponding author, email: assaf.friedler@mail.huji.ac.il

**Table S1: The iASPP-derived peptides in the array ***

**Peptide iASPP Pro peptide sequence**

**Spot residues**

A7 3-18 EAFQSARDFLDMNFQ

A8 12-26 FLDMNFQSLAMKHMD

A9 20-34 LAMKHMDLKQMELDT

A10 28-42 KQMELDTAAAKVDEL

A11 36-50 AAKVDELTKQLESLW

A12 44-58 KQLESLWSDSPAPPG

A13 52-66 DSPAPPGPQAGPPSR

A14 60-74 QAGPPSRPPRYSSSS

A15 68-82 PRYSSSSIPEPFGSR

A16 76-90 PEPFGSRGSPRKAAT

A17 84-98 SPRKAATDGADTPFG

A18 92-106 GADTPFGRSESAPTL

A19 100-114 SESAPTLHPYSPLSP

A20 108-122 PYSPLSPKGRPSSPR

A21 116-130 GRPSSPRTPLYLQPD

A22 124-138 PLYLQPDAYGSLDRA

A23 132-146 YGSLDRATSPRPRAF

A24 140-154 SPRPRAFDGAGSSLG

B1 148-162 GAGSSLGRAPSPRPG

B2 156-170 APSPRPGPGPLRQQG

B3 164-178 GPLRQQGPPTPFDFL

B4 172-186 PTPFDFLGRAGSPRG

B5 180-194 RAGSPRGSPLAEGPQ

B6 188-202 PLAEGPQAFFPERGP

B7 196-210 FFPERGPSPRPPATA

B8 204-218 PRPPATAYDAPASAF

B9 212-226 DAPASAFGSSLLGSG

B10 220-234 SSLLGSGGSAFAPPL

B11 228-242 SAFAPPLRAQDDLTL

B12 236-250 AQDDLTLRRRPPKAW

B13 244-258 RRPPKAWNESDLDVA

B14 252-266 ESDLDVAYEKKPSQT

B15 260-274 EKKPSQTASYERLDV

B16 268-282 SYERLDVFARPASPS

B17 276-290 ARPASPSLQLLPWRE

B18 284-298 QLLPWRESSLDGLGG

B19 292-306 SLDGLGGTGKDNLTS

B20 300-314 GKDNLTSATLPRNYK

B21 308-322 TLPRNYKVSPLASDR

B22 316-330 SPLASDRRSDAGSYR

B23 324-338 SDAGSYRRSLGSAGP

B24 332-346 SLGSAGPSGTLPRSW

C1 340-354 GTLPRSWQPVSRIPM

C2 348-362 PVSRIPMPPSSPQPR

C3 356-370 PSSPQPRGAPRQRPI

C4 364-378 APRQRPIPLSMIFKL

C5 372-386 LSMIFKLQNAFWEHG

C6 380-394 NAFWEHGASRAMLPG

C7 388-402 SRAMLPGSPLFTRAP

C8 396-410 PLFTRAPPPKLQPQP

C9 404-418 PKLQPQPQPQPQPQS

C10 412-426 PQPQPQSQPQPQLPP

C11 420-434 PQPQLPPQPQTQPQT

C12 428-442 PQTQPQTPTPAPQHP

C13 436-450 TPAPQHPQQTWPPVN

C14 444-458 QTWPPVNEGPPKPPT

C15 452-466 GPPKPPTELEPEPEI

C16 460-474 LEPEPEIEGLLTPVL

C17 468-482 GLLTPVLEAGDVDEG

C18 476-490 AGDVDEGPVARPLSP

C19 484-498 VARPLSPTRLQPALP

C20 492-506 RLQPALPPEAQSVPE

C21 500-514 EAQSVPELEEVARVL

C22 508-522 EEVARVLAEIPRPLK

C23 512-526 RVLAEIPRPLKRRGS

C24 516-530 EIPRPLKRRGSMEQA

D1 524-538 RGSMEQAPAVALPPT

D2 532-546 AVALPPTHKKQYQQI

D3 540-554 KKQYQQIISRLFHRH

D4 548-562 SRLFHRHGGPGPGGP

D5 556-570 GPGPGGPEPELSPIT

D6 564-578 PELSPITEGSEARAG

D7 572-586 GSEARAGPPAPAPPA

D8 580-594 PAPAPPAPIPPPAPS

D9 588-602 IPPPAPSQSSPPEQP

D10 596-610 SSPPEQPQSMEMRSV

D11 604-618 SMEMRSVLRKAGSPR

D12 612-626 RKAGSPRKARRARLN

D13 617-631 AGSPRKARRARLNPL

E1 602-616 PQSMEMRSVLRKAGS

E2 613-627 KAGSPRKARRARLNP

E3 620-634 ARRARLNPLVLLLDA

E4 631-645 LLDAALTGELEVVQQ

E5 638-652 GELEVVQQAVKEMND

E6 649-663 EMNDPSQPNEEGITA

E7 656-670 PNEEGITALHNAICG

E8 667-681 AICGANYSIVDFLIT

E9 674-688 SIVDFLITAGANVNS

E10 685-699 NVNSPDSHGWTPLHC

E11 692-706 HGWTPLHCAASCNDT

E12 703-717 CNDTVICMALVQHGA

E13 710-724 MALVQHGAAIFATTL

E14 721-735 ATTLSDGATAFEKCD

E15 728-742 ATAFEKCDPYREGYA

E16 739-753 EGYADCATYLADVEQ

E17 746-760 TYLADVEQSMGLMNS

E18 757-771 LMNSGAVYALWDYSA

E19 764-778 YALWDYSAEFGDELS

E20 775-789 DELSFREGESVTVLR

E21 782-796 GESVTVLRRDGPEET

E22 793-807 PEETDWWWAALHGQE

E23 800-814 WAALHGQEGYVPRNY

E24 814-828 YFGLFPRVKPQRSKV

* The peptides that showed binding in the peptide array screening are marked in yellow. See main text for details.
